# Deep learning-based classification system of bacterial keratitis and fungal keratitis using anterior segment images

**DOI:** 10.3389/fmed.2023.1162124

**Published:** 2023-05-18

**Authors:** Yeo Kyoung Won, Hyebin Lee, Youngjun Kim, Gyule Han, Tae-Young Chung, Yong Man Ro, Dong Hui Lim

**Affiliations:** ^1^Department of Ophthalmology, Samsung Medical Center, Sungkyunkwan University School of Medicine, Seoul, Republic of Korea; ^2^Department of Electrical Engineering, Korea Advanced Institute of Science and Technology, Daejeon, Republic of Korea; ^3^Department of Digital Health, Samsung Advanced Institute for Health Sciences and Technology, Sungkyunkwan University, Seoul, Republic of Korea

**Keywords:** anterior segment image, bacterial keratitis, convolutional neural network (CNN), deep learning (DL), fungal keratitis, infectious keratitis, lesion guiding module (LGM), mask adjusting module (MAM)

## Abstract

**Introduction:**

Infectious keratitis is a vision threatening disease. Bacterial and fungal keratitis are often confused in the early stages, so right diagnosis and optimized treatment for causative organisms is crucial. Antibacterial and antifungal medications are completely different, and the prognosis for fungal keratitis is even much worse. Since the identification of microorganisms takes a long time, empirical treatment must be started according to the appearance of the lesion before an accurate diagnosis. Thus, we developed an automated deep learning (DL) based diagnostic system of bacterial and fungal keratitis based on the anterior segment photographs using two proposed modules, Lesion Guiding Module (LGM) and Mask Adjusting Module (MAM).

**Methods:**

We used 684 anterior segment photographs from 107 patients confirmed as bacterial or fungal keratitis by corneal scraping culture. Both broad- and slit-beam images were included in the analysis. We set baseline classifier as ResNet-50. The LGM was designed to learn the location information of lesions annotated by ophthalmologists and the slit-beam MAM was applied to extract the correct feature points from two different images (broad- and slit-beam) during the training phase. Our algorithm was then externally validated using 98 images from Google image search and ophthalmology textbooks.

**Results:**

A total of 594 images from 88 patients were used for training, and 90 images from 19 patients were used for test. Compared to the diagnostic accuracy of baseline network ResNet-50, the proposed method with LGM and MAM showed significantly higher accuracy (81.1 vs. 87.8%). We further observed that the model achieved significant improvement on diagnostic performance using open-source dataset (64.2 vs. 71.4%). LGM and MAM module showed positive effect on an ablation study.

**Discussion:**

This study demonstrated that the potential of a novel DL based diagnostic algorithm for bacterial and fungal keratitis using two types of anterior segment photographs. The proposed network containing LGM and slit-beam MAM is robust in improving the diagnostic accuracy and overcoming the limitations of small training data and multi type of images.

## 1. Introduction

Infectious keratitis is a common cause of permanent blindness worldwide and can cause serious complications such as corneal perforation, corneal opacification, and endophthalmitis if not properly treated (1–6). Approximately 2,300,000 cases of microbial keratitis (including those caused by bacteria, fungi, viruses, and *Acanthamoeba*) occur annually in South Korea, where bacteria still dominate as the causative organisms of the disease (5). It is known to show various patterns depending on the region, climate, and country. For example, in temperate climates, fungal and mixed infections are more common than in tropical and semi-tropical areas. From an epidemiological point of view, ocular trauma and contact lens-associated keratitis have been increasing in recent years (7, 8).

The selection of an effective antimicrobial agent requires the identification of the causative microorganism. The gold standard for diagnosis is corneal scraping and culture, but it is not always available, and bacterial or fungal growth on culture plates takes several days or weeks (9–11). Even if it is actually microorganism positive, the result may be negative and the lesion may worsen while waiting for the result. Therefore, empirical therapy with broad-spectrum antibiotics, antifungals, and antiviral agents should be initiated based on the clinical experience of the ophthalmologist, based on the shape, size, depth, and location of the lesion, before culture results are obtained (9–12). However, bacterial and fungal keratitis are not completely distinct from each other. If patients receive unnecessary or late treatment due to an incorrect diagnosis, it may result in poor outcomes for the sufferer’s vision, poor quality of life, and increased medical expenses.

Because the deep learning approach has shown remarkable performance in various image processing tasks such as classification and object detection, it has been applied in numerous research fields. Deep learning, using various types of medical images, is also used for the accurate diagnosis and treatment of many ocular diseases. As a result of the development of the methods based on deep learning, the diagnostic performance has been equivalent to or even surpassed the diagnostic ability of clinicians (13–15). Therefore, we expect that the application of deep learning in keratitis diagnosis can assist clinicians in reducing misdiagnoses and improving medical equity and accessibility to medical care.

In this context, we propose a deep learning-based computer-aided diagnosis (CAD) network that classifies and diagnoses bacterial and fungal keratitis combining with two novel modules which can improve keratitis diagnosis accuracy and predict more accurate lesion areas than conventional models.

## 2. Materials and methods

### 2.1. Study approval

This study was performed at Samsung Medical Center (SMC) and Korea Advanced Institute of Science and Technology (KAIST) according to the tenets of the Declaration of Helsinki. The Institutional Review Board of SMC (Seoul, Republic of Korea) approved this study (SMC 2019-01-014).

### 2.2. Participants and data collection

A retrospective analysis of the medical records of patients who had been diagnosed and treated for infectious keratitis (bacterial and fungal keratitis) at the SMC between January 1, 2002, and December 31, 2018, was conducted. All the patients underwent corneal scraping and culture; other forms of keratitis, such as viral or acanthamoeba keratitis, were excluded in this study.

**FIGURE 1:**
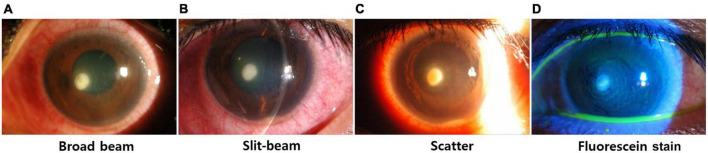
Examples of various types of anterior segment images. **(A)** Broad-beam image, **(B)** slit-beam image, **(C)** scatter image, **(D)** fluorescein stain.

Anterior segment image dataset, called the SMC dataset, is a set of anterior segment images collected from 107 patients. It consists of broad-beam and slit-beam anterior segment images (Figure 1) (16). A total of 594 images from 88 patients were collected for training splits, and 90 images from 19 patients were collected for the test split. The training set comprised 361 images of 64 bacterial keratitis and 233 images of 24 fungal keratitis. The test set comprised 46 images of 13 bacterial keratitis and 24 images of 6 fungal keratitis. None of the patients belonged to both the training and test splits simultaneously. For the experiment, each image was resized to 500 pixels × 750 pixels. Three ophthalmologists (Y.K., T-YC., and D.H.L.) annotated lesions on images related to the diagnosis of keratitis.

To verify the performance of the proposed network, we made the open source dataset consisted of 98 anterior segment images which were collected from Google image search and ophthalmology textbooks (17–19), and used it only as a test split.

The distribution of the images is shown in Table 1.

**TABLE 1:** The distribution of images in SMC dataset and open source dataset.

	SMC dataset				Open source dataset	
	Training split		Test split		Test split	
	Broad-beam	Slit-beam	Broad-beam	Slit-beam	Broad-beam	Slit-beam
Bacterial keratitis	149	212	25	21	58	4
Fungal keratitis	99	134	19	25	32	4

SMC, Samsung Medical Center.

### 2.3. Proposed network

**FIGURE 2:**
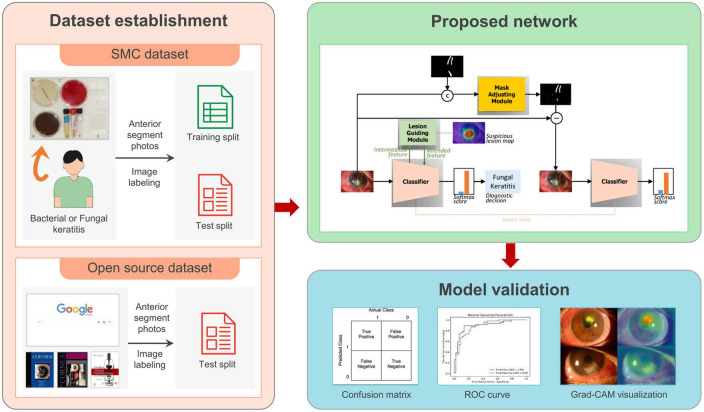
An overview of the entire study design and the proposed network framework.

An overview of the entire study design and the proposed network framework is shown in Figure 2. It contains two proposed modules: the lesion guiding module (LGM) and slit-beam mask adjusting module (MAM). Each module was attached to the main classifier. These two modules were introduced to overcome the aforementioned limitations for classifying the cause of keratitis. In the training stage, LGM makes the network attend to the lesion instead of other details in the anterior segment image, such as reflected light. Because its output has the form of a heat map, the detected lesion location can be obtained. MAM finely generates an optimal mask-pointing slit-beam and small parts that have less impact on the diagnosis. By comparing the masked and unmasked input images in the learning process, the network distinguishes between the necessary and unnecessary parts for diagnosis in the anterior segment image and acquires the ability to not pay attention to the unnecessary parts. We set the baseline classifier to ResNet-50 (15), and the architecture of our proposed network was based on ResNet-50. Three LGMs were inserted between each residual block of the ResNet-50.

#### 2.3.1. LGM

**FIGURE 3:**
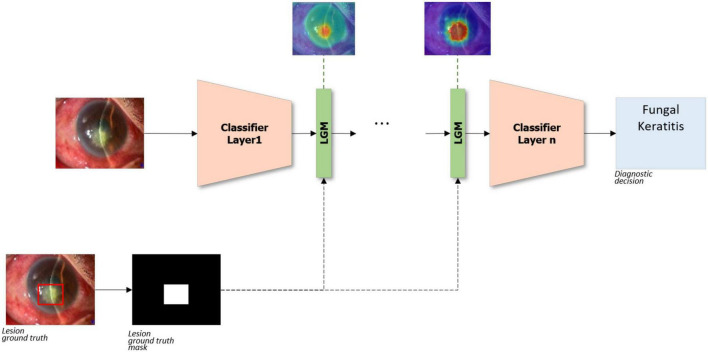
Lesion guiding modules applied on classifier. LGM, lesion guiding module.

Lesion Guiding Module is designed for deep learning-based diagnostic systems to learn the location information of lesions annotated by ophthalmologists in the anterior segment image. In a classifier with convolutional layers, the *n*LGMs are inserted between the layers, as shown in Figure 3. The bounding boxes annotated by ophthalmologists are converted to a binary mask before being input to LGM. In LGM, the intensity of the intermediate feature maps is multiplied with a binary mask during the training stage. Following this process, an important part of the diagnosis has a negative value and a relatively unrelated part of the diagnosis has a positive value. Therefore, LGM is trained to minimize the loss function and can point to lesions that are correlated to the diagnosis.

#### 2.3.2. Slit-beam MAM

**FIGURE 4:**
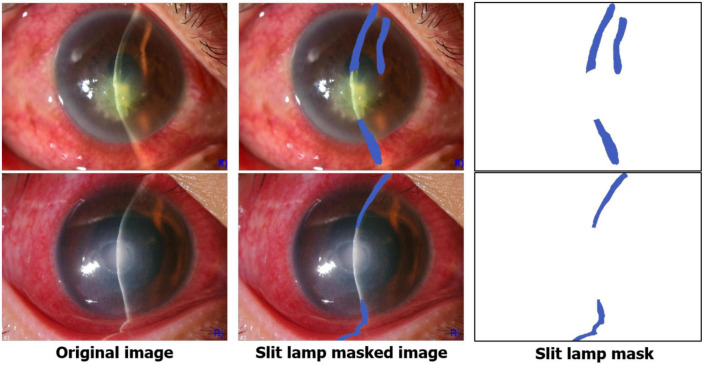
Examples of the slit-beam mask annotation.

In contrast to LGM learning information about areas to be focused on, the slit-beam MAM learns information about areas that should not be focused on. By using MAM in a training phase, the single network can efficiently learn slit-beam images and broad-beam images without paying attention to the slit-beam portion of the slit-beam image (Figure 4). In addition, it can prevent the network from focusing on complex textures, such as eyelashes or blood vessels, in the anterior segment image. MAM is a module that allows the main classification network to focus on the important parts for diagnosis, so it is used only in the training phase and not in inference.

Details of the proposed network and training procedure are provided in the Supplementary material and Supplementary Figures 1–3.

### 2.4. Evaluation metrics

We compared the diagnostic performance of our deep learning network system with that of the baseline classifier, ResNet-50. To evaluate the accuracy of the diagnosis, the simple accuracy when the cause had the higher probability score was assumed as the final decision and areas under the receiver operating characteristic curve (AUC) were calculated. In addition, the accuracy of the detected lesion location during the diagnosis process was measured using the intersection over union (IOU) metric. The IOU is the ratio of the overlap between the predicted bounding box and the ophthalmologist’s manually labeled bounding box. The closer the IOU value is to 1, the more accurate is the location of the detected lesion. We also visualized the spatial attention map of LGM. To verify the individual effects of the proposed modules on diagnostic accuracy, we conducted an ablation study.

## 3. Results

### 3.1. Performance of the metrics

Details of the performance of baseline (ResNet-50) and the proposed method are shown in Table 2. The proposed method showed the higher values in all classification performances including accuracy than baseline with both SMC dataset and open source dataset.

**TABLE 2:** Classification performance of baseline and proposed model.

Networks	Dataset	Classification performance				
		Sensitivity	Specificity	Accuracy	Precision	F1 score
Baseline (Resnet-50)	SMC dataset	0.750	0.870	0.811	0.784	0.825
	Open source dataset	0.333	0.823	0.643	0.680	0.745
Proposed method	SMC dataset	0.864	0.891	0.878	0.872	0.882
	Open source dataset	0.417	0.887	0.714	0.724	0.797

SMC, Samsung Medical Center.

### 3.2. Diagnostic accuracy

By inferencing the two datasets, we obtained the diagnostic accuracy, as shown in Table 3. Comparing the diagnostic accuracy of the baseline network when the training image type was only a broad-beam and both a broad-beam and slit-beam, the accuracy of the broad-beam image decreased even though the number of images was more than doubled (B:0.818 → B:0.795). This result means that for different image types, simply increasing the number of images does not work properly to improve the performance. Furthermore, the network trained with broad-beam images show low diagnostic accuracy in slit-beam images (S:0.587).

**TABLE 3:** Diagnostic accuracy on dataset.

Networks	Image types used in training	Accuracy	
		SMC dataset	Open source dataset
Baseline (Resnet-50)	Broad-beam	0.700 (B:0.818/S:0.587)	0.653
	Broad-beam, Slit-beam	0.811 (B:0.795/S:0.826)	0.642
Proposed method	Broad-beam, Slit-beam	0.878 (B:0.909/S:0.848)	0.714

SMC, Samsung Medical Center. B represents accuracy for only broad-beam images and S represents accuracy for only slit-beam images.

In contrast, the network with the proposed modules showed an approximately 8% increase in the SMC dataset and 7% in the open source dataset on diagnostic accuracy compared to those of the baseline network trained with both a broad-beam and slit-beam images.

### 3.3. Lesion localization

Supplementary Figure 4 showed the calculated IOU with various thresholds. Among these values, the best value occurred when the threshold was 0.45, as shown in Table 4. This shows that the spatial attention map of LGM, which was located between ResNet blocks 3 and 4, was more accurate than the baseline network with Grad-CAM (20).

**TABLE 4:** Localization performance with IOU metric on the SMC dataset.

Networks	Localization method	Mean IOU (Threshold = 0.45)
Baseline (ResNet-50)	Grad-CAM	0.175
Proposed method	LGM (between ResNet layer 3 and 4)	0.489

IOU, intersection over union; SMC, Samsung Medical Center; Grad-CAM, gradient-weighted class activation mapping; LGM, lesion guiding module.

### 3.4. Ablation study

**FIGURE 5:**
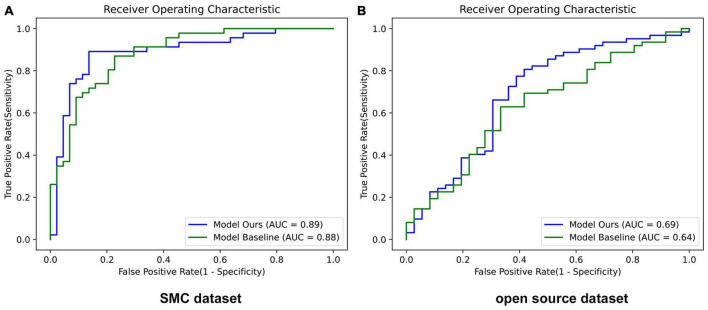
Performance of ROC curves in baseline and our proposed network. **(A)** SMC dataset and **(B)** Open source dataset. Model Ours is a new deep learning model that combines LGM and MAM with the baseline ResNet-50. Model Baseline is ResNet-50. AUC, area under the curve.

Table 5 showed the results demonstrating that LGM and MAM modules had a positive effect on the accuracy of the SMC dataset and open source dataset both. Corresponding ROC curves are shown in Figure 5.

**TABLE 5:** Ablation study on dataset.

Networks	Accuracy	
	SMC dataset	Open source dataset
Baseline (ResNet-50)	0.811 (B:0.795/S:0.826)	0.642
Baseline + LGM	0.856 (B:0.864/S:0.848)	0.673
Baseline + MAM	0.833 (B:0.841/S:0.826)	0.653
Baseline + LGM + MAM	0.878 (B:0.909/S:0.848)	0.714

B represents accuracy for only broad-beam images and S represents accuracy for only slit-beam images. LGM, lesion guiding module; MAM, mask adjusting module; SMC, Samsung Medical Center.

### 3.5. Qualitative results

**FIGURE 6:**
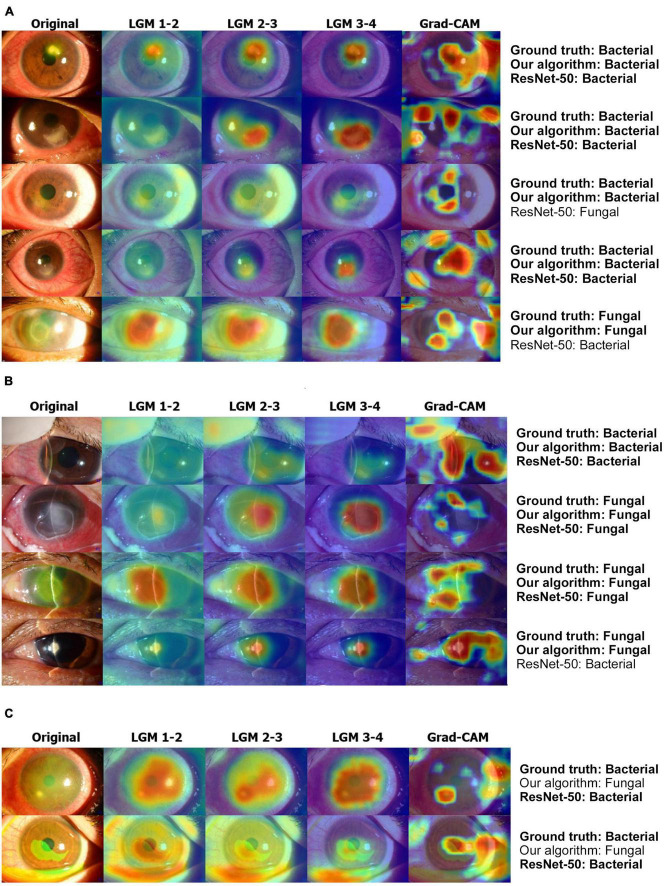
Examples of result visualization. **(A)** Broad-beam images, **(B)** slit-beam images, and **(C)** misdiagnosis cases. LGM 1-2 denotes LGM between ResNet blocks 1 and 2. LGM 2-3 denotes LGM between ResNet blocks 2 and 3. LGM 3-4 denotes LGM between ResNet blocks 3 and 4. LGM, Lesion guiding module; Grad-CAM, gradient-weighted class activation mapping.

Figure 6A showed that LGM points to more accurate lesion areas regardless of lesion size or shape, whereas the Grad-CAM method has high values for complex textures such as eyelashes and blood vessels (20). LGM shows that it does not attend to the reflected light in the anterior segment images. In the case of the slit-beam images, the Grad-CAM method tends to point not only to lesions but also to slit-beam (20), whereas LGM only attends to lesions without adjusting the slit-beam (Figure 6B). In the case of misdiagnosed images, as shown in Figure 6C, most of the anterior segment images were obtained from the stained eyes. At this time, it could be shown that LGM tends to point to an excessively wide area, including a lesion site or an area unrelated to diagnosis.

## 4. Discussion

We developed a novel deep learning algorithm that specializes in diagnosing bacterial and fungal keratitis by analyzing anterior segment images. A representative convolutional neural network (CNN), ResNet-50, was used as the backbone of the algorithm with the two proposed modules, LGM and MAM, resulting in high performance in distinguishing the images with bacterial keratitis from those with fungal keratitis.

The early and accurate diagnosis of infectious keratitis is essential for resolving the infection and minimizing corneal damage (12). Generally, the presence of an irregular/feathery border, satellite lesions, and endothelial plaque is associated with fungal keratitis, whereas a wreath infiltrate or epithelial plaque is associated with bacterial keratitis (21). However, it is difficult to distinguish exactly based on the characteristics of the infected lesions. Bacterial and fungal keratitis are often confused, especially in the early stages, but the medications used are different, and the prognosis for fungal keratitis is much worse (22–24). If patients with keratitis receive unnecessary or late treatment due to an incorrect diagnosis, complications such as corneal opacity, poor outcome for vision, or endophthalmitis may occur. Therefore, we focused on differentiating between bacterial and fungal keratitis among patients with infectious keratitis in this study.

A deep-learning approach was used to analyze a variety of medical images. Recent advances in deep learning technology in the ophthalmic field have also allowed rapid and accurate diagnosis of several ocular diseases (14, 25). However, unlike deep learning systems using optical coherence tomography and retinal fundus images, only a few studies dealing with anterior segment images have been published. In particular, in the case of infectious keratitis, it is difficult to apply a deep learning algorithm directly because it is related to lesions in various positions, and it is difficult to identify the causative pathogen without corneal culture.

In this study, we utilized a deep learning CAD network system for the differential diagnosis of bacterial and fungal keratitis based on the different shapes of corneal lesions. There are two major challenges in diagnosing the cause of keratitis by using anterior segment images. First, because of the corneal aspheric shape, the depth and extent of the infiltration lesions that were seen in the actual slit-lamp examination are not clearly visible in the image, and small lesions are often not represented in the image. Additional information such as trauma (dirty water, soil, soft contact lens, etc.) or past medical history of the patient are very important for diagnosis. Furthermore, the appearance differs depending on the light source; therefore, keratitis is often misdiagnosed. Therefore, for an accurate diagnostic network, the knowledge of experienced ophthalmologists who can distinguish the lesion related to the diagnosis from other anatomical parts should be transferred to the diagnostic network. Second, it is difficult to obtain a large number of anterior segment images of keratitis from the various aspects of each pathogen. It is also problematic that anterior segment images can be captured in different ways. These diverse features make it difficult to learn a diagnosis network with a finite number of weights, reducing diagnostic accuracy. To solve these problems, a method for learning the features regardless of the type of anterior segment image is required. We propose two modules to solve these two challenges.

The two proposed modules, LGM and MAM, were combined with the main classifier. In the feature extraction process, LGM attends to a suspicious area related to diagnosis. Because its output has the form of a heat map, the detected lesion location can be obtained. The slit-beam MAM is used only in the training stage; it generates an optimal mask pointing to the slit-beam and small parts that have less impact on diagnosis. The unnoticed part of the actual diagnosis process is also suppressed in the learning process of the network so that the anterior segment images with and without the slit-beam can be effectively learned together. Through training a network using this module in the proposed procedure, the network can learn different types of anterior segment image (broad-beam and slit-beam) efficiently, and we obtained a high diagnostic accuracy for infectious keratitis using different types of images.

Similar to our deep learning model, some recent other studies have applied deep learning models to distinguish patients with fungal keratitis from those with bacterial keratitis using anterior segment images. The algorithms by Hung et al. (26) based on DenseNet161 and ResNet-50 achieved average accuracies of 0.786 and 0.773, respectively. Ghosh et al. (27) constructed a model called deep keratitis based on ResNet-50 with Grad-CAM. However, its precision was 0.57 (95% CI: 0.49−0.65) which was lower than 0.878 in our results. The model with VGG19 exhibited the highest performance (0.88). Redd et al. (28) showed the highest AUC of 0.86 in MobileNet among 5 CNNs, which was nearly similar with our AUC result (0.89). Our model achieved an overall accuracy of approximately 88%, which is comparable to these previous models. Most previous studies just emphasized the application of deep learning techniques for the diagnosis of infectious keratitis. Rather than suggesting a new developed deep learning network, they analyzed the performance of each existing CNN such as ResNet, DenseNet, and ResNeXt in diagnosing the keratitis. Meanwhile, to our knowledge, we first presented a novel deep learning framework combined with two proposed modules, which is specialized in diagnosing bacterial and fungal keratitis. Furthermore, several studies have been published recently to distinguish the infectious keratitis by causative pathogens including bacterial, fungal, acanthamoeba, or viral keratitis. Zhang et al. (29) and Koyama et al. (14) provided the deep learning based diagnostic models for 4 types of infectious keratitis, and they also showed the lower accuracy in diagnosing bacteria and fungi than acanthamoeba or virus.

This study had several limitations. First, we only distinguished between bacterial and fungal keratitis. Indeed, the causes of infectious keratitis are diverse, and it is difficult to discriminate non-infectious immune keratitis in the early stages. Through subsequent studies, we need to develop a system to discriminate between the various types of keratitis. Second, the training process of LGM requires a hand-labeled bounding box annotation by specialists who are experienced in keratitis diagnosis. Therefore, a significant amount of time and resources are required to train the model. In MAM, a sophisticated pixel-level slit-beam mask is required for learning. Relatively less expertise is required than for lesion annotation, but it is also difficult to obtain masks in large quantities because of the higher accuracy required for pixel-level labeling than for bounding boxes. The expensive work required to obtain resources for learning can be a deterrent preventing the proposed network from being used in practice. Third, the usability of LGM is limited. The broad-beam anterior segment images with and without the slit-beam used in this experiment had a relatively high similarity to each other compared to other types (scatter and stain images). Therefore, by applying LGM to the original and image-level modified image, effective learning of the diagnostic system could be achieved regardless of the presence of the slit-beam in this study. However, to apply a similar mechanism to all types of anterior segment images, a method of dividing image features into parts having diagnostic information at the feature level and parts that are unnecessary for diagnosis is required. Fourth, although the LGM technique of our study allowed us to distinguish between bacterial keratitis and fungal keratitis by first accurately finding the lesion and looking at the characteristics of the lesion, exactly which part of the lesion was used to distinguish between bacterial and fungal keratitis is unknown with the results. However, LGM can identify the pathologic areas accurately by focusing only on lesions without any other noise than the Grad-Cam in the heatmaps. Finally, we combined our two modules with ResNet-50. Because some previous deep learning algorithms tend to show higher performance in other CNNs, not on ResNet-50, further studies comparing different CNNs applying our modules are required to enhance the diagnostic accuracy of infectious keratitis.

In conclusion, our deep-learning framework for the diagnosis of infectious keratitis was successfully developed and validated. LGM is presented for an accurate diagnosis by emphasizing the lesions associated with the diagnosis. To prevent the less-informative part from affecting the diagnostic result and to efficiently learn two different types of anterior segment images in a single network, we designed a new learning procedure using a masking module, MAM, to control masking in the training phase. The results showed that our proposed module had a meaningful effect in enhancing the diagnostic performance of bacterial and fungal keratitis on different anterior segment image datasets.

## Data availability statement

The data analyzed in this study is subject to the following licenses/restrictions: The approval process from research data review committee is required and the dataset is not open to the public. Requests to access these datasets should be directed to YW, wyk900105@hanmail.net.

## Ethics statement

This study was performed at Samsung Medical Center (SMC) and Korea Advanced Institute of Science and Technology (KAIST) according to the tenets of the Declaration of Helsinki. The Institutional Review Board of SMC (Seoul, Republic of Korea) approved this study (SMC 2019-01-014).

## Author contributions

DL and YR designed the study, reviewed the design and results, and submitted the draft. YK, T-YC, and DL annotated lesions on images related to the diagnosis of keratitis. YW, HL, YK, and GH analyzed and interpreted the clinical data. YW and HL drafted the submitted manuscript draft. All authors have read and approved the final manuscript.
