# Supplementary material for: Deep learning-based classification system of bacterial keratitis and fungal keratitis using anterior segment images
Source: Front Med (Lausanne). 2023 May 18;10:1162124. doi: 10.3389/fmed.2023.1162124 (PMC10233039; doi:10.3389/fmed.2023.1162124)
Supplement: Supplementary file 1 [file Table_1.DOCX]

***Supplementary Material***

**Deep Learning-based Classification System of Bacterial Keratitis and Fungal Keratitis using Anterior Segment Images**

**Yeo Kyoung Won^1*^, Hyebin Lee^2*^, Youngjun Kim^1^, Gyule Han^1^, Tae-Young Chung^1^, Yong Man Ro^2†^, Dong Hui Lim^1,3†^**

^1^ Department of Ophthalmology, Samsung Medical Center, Sungkyunkwan University School of Medicine, Seoul, Republic of Korea

^2^ Department of Science in Electrical Engineering, Korea Advanced Institute of Science and Technology, Daejeon, Republic of Korea

^3^ Department of Digital Health, Samsung Advanced Institute for Health Sciences and Technology, Sungkyunkwan University, Seoul, Republic of Korea

All authors declare that they have no conflict of interest.

*Corresponding authors:

Dong Hui Lim, MD, PhD

Department of Ophthalmology, Samsung Medical Center, Sungkyunkwan University School of Medicine, Seoul 06351, Republic of Korea

Tel: 82-2-3410-3548, Fax: 82-2-3410-0074, E-mail: [ldhlse@gmail.com](mailto:ldhlse@gmail.com)

**Yong Man Ro, PhD**

Department of Science in Electrical Engineering, Korea Advanced Institute of Science and Technology, Daejeon 34141, Republic of Korea

Tel: 82-42-350-3494, Fax: 82-42-350-5494, E-mail: ymro@kaist.ac.kr

*These authors contributed equally as co-first authors.

†These authors contributed equally to this article as co-corresponding authors.

1. Details of the Deep Learning Algorithm
   1. Architecture

An overview of the proposed network framework is shown in Figure 2. It contains two proposed modules, which are the Lesion Guiding Module (LGM) and the slit lamp Mask Adjusting Module (MAM). Each module is attached to the main classifier. Unlike LGM, which is used for both training phase and test phase, MAM is a module that helps efficient learning of multiple images and is used only for the training phase.

Lesion Guiding Module

The Lesion Guiding Module (LGM) is a module designed for deep learning-based diagnostic systems to learn the location information of lesions annotated by ophthalmologists in the anterior segment image. In a classifier with convolutional layers, the $n$ LGMs are inserted between the layers, as shown in Figure 3. LGM takes intermediate feature maps from the classfier $\mathbf{f}_{n}^{cls}$ sized $h_{n}\times w_{n}\times c_{n}$ as input.

In LGM in Supplementary Figure 1, input $\mathbf{f}_{n}^{cls}$ converted as a form of single channel spatial attention $\mathbf{S}_{n}$ sized $h_{n}\times w_{n}$.

This process inspired by BAM [1]. The reduction ratio and dilation value are set to 16 and 4, same as BAM. Detailed operation to get $\mathbf{S}_{n}$ is

| $\mathbf{S}_{n}=\text{sigmoid}(BN(f_{3}^{1\times1}(f_{2}^{3\times3}(f_{1}^{3\times3}(f_{0}^{1\times1}(\mathbf{f}_{n}^{cls}))))))$, | (1) |
| --- | --- |

where $f^{k\times k}$ denotes convolutional layer with $k\times k$ filter and $BN(\cdot)$ denotes a batch normalization. Lesion attended feature from $n\text{-th}$ LGM $\mathbf{f}_{n}^{lesion}$ is generated by combine $\mathbf{f}_{n}^{cls}$ and $\mathbf{S}_{n}$as a form of residual attention

| $\mathbf{f}_{n}^{lesion}(n,m,k)=\mathbf{f}_{n}^{cls}(n,m,k)\odot\mathbf{S}_{n}(n,m)+\mathbf{f}_{n}^{cls}(n,m,k)$, | (2) |
| --- | --- |

where $\odot$ denotes element-wise multiplication. $\mathbf{f}_{n}^{lesion}$ is used as input of n+1-th layer of classifier.

In order to train LGM, Spatial attention ground truth $\mathbf{S}_{n}^{GT}$ sized $h_{n}\times w_{n}$ is used. $\mathbf{S}_{n}^{GT}$ is a kind of mask which has value -1 for the location of lesion and other parts of mask is 1. $\mathbf{S}_{n}^{GT}$ can be obtained by resizing ophthalmologist’ annotation map which is the same size of original anterior segment image input. Lesion guiding loss $\mathcal{L}_{LGM}$ is calculated as

| $\mathcal{L}_{LGM}=\sum_{n} \frac{\mathbf{S}_{n}^{GT}\odot\mathbf{S}_{n}}{h_{n}\times w_{n}} .$ | (3) |
| --- | --- |

According to equation 3, if $\mathbf{S}_{n}$ has large value on non-lesion part, $\mathcal{L}_{LGM}$ increases. Contrary, $\mathbf{S}_{n}$ has large value on lesion part, $\mathcal{L}_{LGM}$ decreases.

Slit-Beam Mask Adjusting Module

The purpose of the slit-beam Mask Adjusting Module (MAM) is to extract the correct feature points from two different types of anterior segment image inputs (broad bream, slit-beam) during the training phase. Since the number of learnable weights of the deep learning based classifier is limited, the capacity for learning various feature is also limited. As a result, more diverse image types and feature points make classification more difficult. To deal with this limitation and make the training process efficiently for the multi-type image, MAM transfer the prior knowledge that the specific part which is not important to make the decision to the network.

In order to train with MAM, slit beam mask $\mathbf{M}$ is required such as shown in Figure 4. Slit beam masking is pixel-wise binary mask which is contain location of slit beam on the image excluding lesion parts. The pixel value of $\mathbf{M}$ corresponding slit beam is 1 and other part is 0. MAM takes $\mathbf{M}$**’s** corresponding anterior segment image $\mathbf{I}$ as the input. MAM has two branch-generating binary masks: $\mathbf{M}_{pos}$ and $\mathbf{M}_{neg}$. $\mathbf{M}_{pos}$ covers an informative area that should not be included in the adjusted mask $\mathbf{M}_{MAM}$**.** In contrast, $\mathbf{M}_{neg}$ covers unnecessary areas that should be included in $\mathbf{M}_{MAM}$**.**

For MAM, concatenated result of original anterior segment image ($\mathbf{I}+\mathbf{M}$**)** and corresponding $\mathbf{M}$ is provided as input. MAM contains two sets of convolutional layers $f_{pos}$ and $f_{neg}$, which make binary mask $\mathbf{M}_{pos}$ and $\mathbf{M}_{neg}$ as

| $\begin{aligned} \mathbf{M}_{pos}=f_{pos}(\mathbf{I+M}) \\ \mathbf{M}_{neg}=f_{neg}(\mathbf{I+M}) \end{aligned}$. | (4) |
| --- | --- |

As shown in Supplementary Figure 2, though addition and subtraction operation, adjusted mask $\mathbf{M}_{MAM}$ is generated as

| $\mathbf{f}_{n}^{lesion}(n,m,k)=\mathbf{f}_{n}^{cls}(n,m,k)\odot\mathbf{S}_{n}(n,m)+\mathbf{f}_{n}^{cls}(n,m,k)$, | (5) |
| --- | --- |

where $\odot$ denotes element-wise multiplication and $\mathbf{J}$ denote matrix of ones which has identical size with $\mathbf{M}$. According to equation 5, $\mathbf{M}_{pos}$ represents the area which mask should contain and $\mathbf{M}_{neg}$ represents the area which mask should exclude.

Supplementary Figure 3 shows procedure to train MAM. Mask difference loss $\mathcal{L}_{M}$ and softmax difference loss $\mathcal{L}_{S}$ are calculated as

| $\mathcal{L}_{M}={\vert\mathbf{M}_{pos}+\mathbf{M}_{neg}\vert}_{1}$, | (6) |
| --- | --- |
| $\mathcal{L}_{S}={\vert S\left( \mathbf{I} \right)-S(\mathbf{I}_{MAM})\vert}_{2}$, | (7) |

where $S(\cdot)$ denotes softmax function and $\mathbf{I}_{MAM}$ denotes slit beam masked anterior segment image. Since $\mathbf{M}_{pos}$ and $\mathbf{M}_{neg}$ always have positive value, $\mathcal{L}_{M}$ is introduced to reduce difference between $\mathbf{M}$ and $\mathbf{M}_{\boldsymbol{MAM}}$ as equation 6. According to equation 7, $\mathcal{L}_{S}$ makes $S\left( \mathbf{I} \right)$ and $S(\mathbf{I}_{MAM})$ similar. During training, MAM tries to find optimal mask which is not that different from $\mathbf{M}$ while two softmax scores $S\left( \mathbf{I} \right)$ and $S(\mathbf{I}_{MAM})$ are similar. At the same time, this process induces the network from focusing on parts other than slit beams, by providing the knowledge that the masked part of the input anterior segment image does not have a significant effect on softmax score and the final diagnostic result.

Training Procedure

Firstly, Classifier with LGM is trained to minimize cross entropy loss $\mathcal{L}_{CE}$ and $\mathcal{L}_{LGM}$:

| $\mathcal{L}_{1}=\mathcal{L}_{CE}(y_{dig},\hat{y}_{dig})+\alpha_{LGM}\mathcal{L}_{LGM}$, | (8) |
| --- | --- |

where $y_{dig}$ and $\hat{y}_{dig}$ denotes final diagnostic result from classifier and ground truth, $\alpha_{LGM}$ is a balancing hyper-parameter. After converging $\mathcal{L}_{1}$ used to train classifier with LGM, MAM is added and train whole network to minimize loss function $\mathcal{L}_{2}$:

| $\mathcal{L}_{2}=\mathcal{L}_{CE}(y_{dig},\hat{y}_{dig})+\alpha_{LGM}\mathcal{L}_{LGM}+\alpha_{M}\mathcal{L}_{M}+\alpha_{S}\mathcal{L}_{S}$, | (9) |
| --- | --- |

where $\alpha_{LGM}$, $\alpha_{M}$ and $\alpha_{S}$ are balancing hyper-parameters and $\mathcal{L}_{M}$ and $\mathcal{L}_{S}$are only calculated when the network input is the slit lamp image.

- 1. Training Details

We set baseline classifier as ResNet-50 [2]. Three LGMs are inserted between each residual block of ResNet-50. The baseline classifier and classifier of the proposed network were initialized with pre-trained weights from model which is trained with ImageNet [3]. In order to increase the number of training images, data augmentation was conducted. The two sizes of patches were cropped from the original image at random locations. Random horizontal flip, vertical flip and random rotation from -10° to 10° were also used. The size of mini-batch was set to 16 and Adam optimizer with learning rate 0.00001 was used. The balancing parameters$\alpha_{LGM}$, $\alpha_{M}$ and $\alpha_{S}$ was set to 1, 1e-5 and 1. All experiments was conducted on a server with 4 TITAN XP GPUs, Intel® Xeon® (E5-2630 v4) CPU, and 128GB memory. We implemented baseline classifier and the proposed model via Pytorch.

**2. Supplementary Figures**

**Supplementary Figure 1.** **Detailed architecture of the lesion guiding module**

$\mathcal{L}_{LGM}, Lesion guiding loss;$ GT, ground truth; $\mathbf{S}_{n};$single channel spatial attention

**
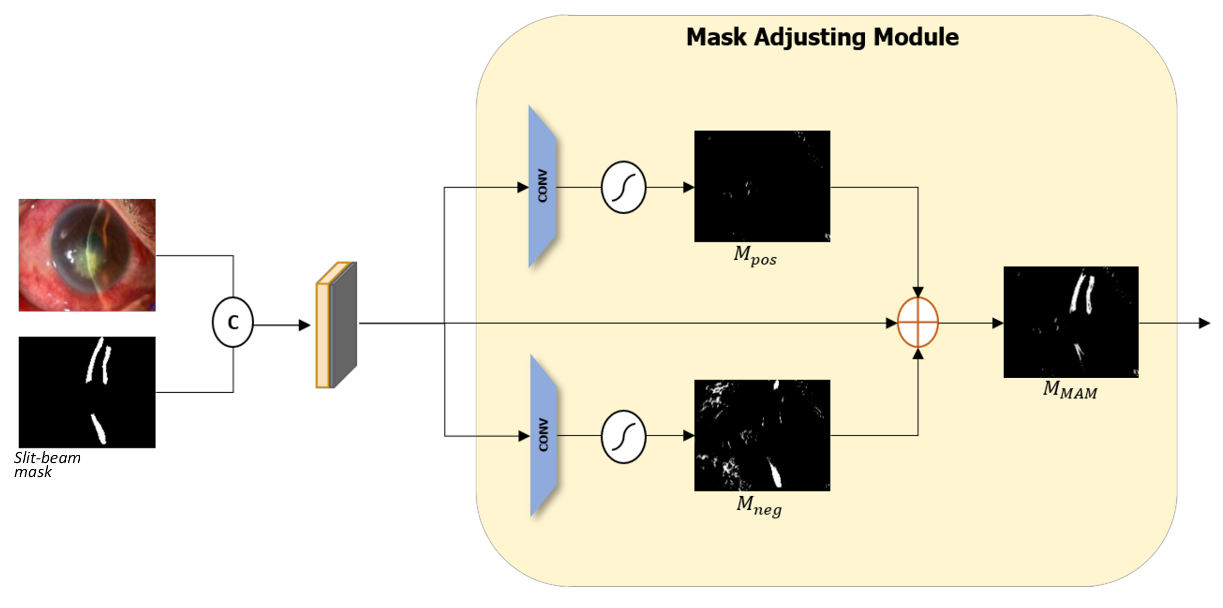
**

**Supplementary Figure 2. Detailed architecture of the mask adjusting module**

$\mathbf{M}_{pos}$ represents the area which mask should contain and $\mathbf{M}_{neg}$ represents the area which mask should exclude. $\mathbf{M}_{pos}$ covers an informative area that should not be included in the adjusted mask $\mathbf{M}_{MAM}$. In contrast, $\mathbf{M}_{neg}$ covers unnecessary areas that should be included in $\mathbf{M}_{MAM}$.

**Supplementary Figure 3. Training procedure with the mask adjusting module**

$\mathcal{L}_{M,}$ Mask difference loss; $\mathcal{L}_{S,}$softmax difference loss


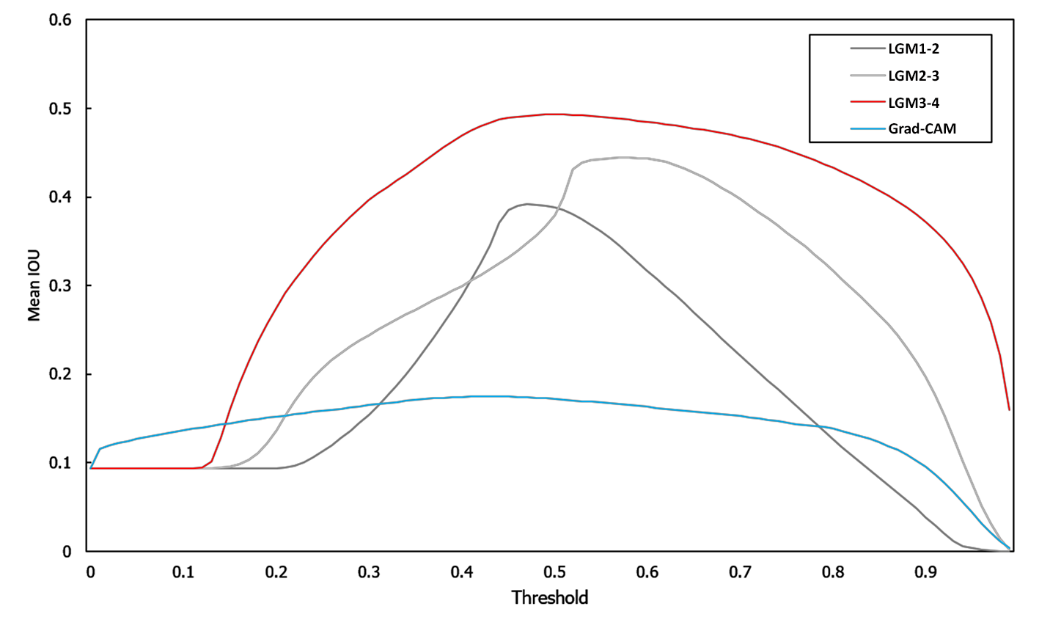


**Supplementary Figure 4. Measured mean IOU values of LGM and Grad-CAM with various thresholds**

LGM 1-2 denotes LGM between ResNet blocks 1 and 2**.** LGM 2-3 denotes LGM between ResNet blocks 2 and 3**.** LGM 3-4 denotes LGM between ResNet blocks 3 and 4**.**

IOU, Intersection over Union; Grad-CAM, Gradient-weighted class activation mapping; LGM, Lesion guiding module

3. References

1. Park, Jongchan, et al. “Bam: Bottleneck attention module.” *British Machine Vision Conference*, 2018
2. He, Kaiming, et al. “Deep residual learning for image recognition.” *Proceedings of the IEEE conference on computer vision and pattern recognition,* 2016.
3. Deng, Jia, et al. “Imagenet: A large-scale hierarchical image database.” *IEEE conference on computer vision and pattern recognition*, 2009
